# Living on a trophic subsidy: Algal quality drives an upper-shore herbivore’s consumption, preference and absorption but not growth rates

**DOI:** 10.1371/journal.pone.0196121

**Published:** 2018-04-19

**Authors:** Diego Quintanilla-Ahumada, Pedro A. Quijón, Jorge M. Navarro, José Pulgar, Cristian Duarte

**Affiliations:** 1 Escuela de Ciencias del Mar, Facultad de Ecología y Recursos Naturales, Universidad Andrés Bello, Santiago, Chile; 2 Coastal Ecology Laboratory, Department of Biology, University of Prince Edward Island, Charlottetown, PE, Canada; 3 Instituto de Ciencias Marinas y Limnológicas, Facultad de Ciencias, Universidad Austral de Chile, Valdivia, Chile; 4 Centro Fondap de Investigación de Ecosistemas Marinos de Altas Latitudes (IDEAL), Valdivia, Chile; 5 Departamento de Ecología y Biodiversidad, Facultad de Ecología y Recursos Naturales, Universidad Andrés Bello, Santiago, Chile; 6 Center for the Study of Multiple-drivers on Marine Socio-Ecological System (MUSELS), Universidad de Concepción, Concepción, Chile; Maurice Lamontagne Institute, CANADA

## Abstract

The transfer of seaweeds from subtidal bottoms to nearby intertidal rocky shores is a common but often overlooked phenomenon. Freshly detached seaweeds often represent critical trophic subsidies for herbivores living in upper-shore rocky intertidal areas, such as the marine snail *Diloma nigerrima*. This species relies on three species of seaweeds for food and displays feeding strategies to deal with a resource that is scarce and at times unpredictable. This study focused on the nutritional quality of freshly detached algae (*Durvillaea antarctica*, *Lessonia spicata* and *Lessonia trabeculata*) and measured *Diloma nigerrima*’s algal consumption rates in trials with and without choice. Absorption efficiency and growth of individual snails fed on each alga were also measured. *Durvillaea antarctica* had the highest nutritional quality and was the most consumed algae in both single and multiple-choice trials. Absorption efficiency was also highest for *D*. *antarctica* but growth rates of snails fed with this species were similar to those fed with the other algae. Combined, these results suggest that *D*. *nigerrima* has the ability to discriminate among seaweeds based on their nutritional quality. A potential increase in oxygen uptake when *D*. *nigerrima* is consuming the preferred food item is also proposed as a plausible hypothesis to explain the mismatch between snails’ preference and growth rate. These results aim to guide further studies on trophic subsidies and their role in coastal systems.

## Introduction

Local-scale processes, such as physical disturbance and species interactions, influence individuals, populations and communities [1–3]. However, separate components of an ecosystem should not be analysed as discrete ecological units [4–6]. Local processes are often affected by external factors as a result of the connectivity between ecotones or ecosystem components [7–9]. Trophic subsidies or the transfer of energy between distinct ecotones [8,10] constitute a prime example of this. Trophic subsidies are widespread in nature but become most relevant when the ecotone receiving the subsidy is naturally poor or deprived of primary producers (e.g. sandy beaches; [11,12]).

Given their proximity, transfer of detritus between subtidal and intertidal rocky shore areas is expected to occur often [13]. On temperate shorelines in particular, where rich subtidal kelp forest are common [14,15] a considerable amount of organic matter associated with detached seaweeds is transferred to intertidal habitats [16–18]. In Southwest Africa, for example, stranded seaweeds coming from subtidal areas support large intertidal populations of *Patella argenvillei* and *Patella granatina*, which in turn “top-down” regulate entire algal communities [19]. Stranded seaweeds also support populations of black sea urchins (*Tetrapygus niger*) in central Chile, which in turn alleviates the pressure of these grazers on the other species of algae in the system [9]. Despite the relevance of subsidies like fresh stranded seaweeds, we still lack a clear understanding of their role on the feeding ecology and fitness of rocky intertidal herbivores (e.g. [20,21])

Herbivores, particularly those relying on trophic subsidies from other systems, are heavily dependent on the quality of the algae they consume [22,23,9]. In comparison to animals, seaweed tissues are low in proteins and some authors have even considered the diet of herbivores to be “protein-limited” [24–27]. This has prompted herbivores to develop physiological or behavioral strategies to fulfill their nutritional requirements in the face of limited or unpredictable food supplies [10]. While some species optimize their diet by choosing algae that are rich in proteins [28,29,10,30], others increase their consumption of lower quality algae (compensatory feeding [31]) or increase protein absorption efficiency [32]. A trade-off of these and other potential strategies is particularly interesting among upper intertidal herbivores such as *Diloma nigerrima*, a marine snail that relies on the supply of freshly detached seaweeds from richer subtidal bottoms along the South American Eastern Pacific [33,34].

On central Chile rocky shores, *Diloma nigerrima* populations reach high densities in upper intertidal areas [33]. This small black snail is globular in shape with indistinct spiral lines, and feeds primarily on three species of freshly stranded algae that grow on subtidal bottoms: *Durvillea antarctica*, *Lessonia spicata* and *Lessonia trabeculata*. The reliance of *D*. *nigerrima* on these algae raises two questions regarding its feeding ecology and fitness. Is this marine snail able to discriminate and choose among algae based on their nutritional quality? And then, is the nutrient absorption efficiency and ultimately snail growth a reflection of potential differences in algal nutritional quality? Two hypotheses are proposed here to address these questions: herbivores such as this snail consume, prefer and achieve the highest absorption efficiency and growth rates on the alga with the best nutritional quality. Alternatively, herbivores do not discriminate among algae and instead balance their growth rates by increasing the absorption efficiency and/or consumption rates on lower quality algae (i.e. compensatory feeding). The first hypothesis is broadly supported by literature in foraging behavior [22,35]. Meanwhile, the absorption efficiency increase or the “compensatory feeding” operating behind the second hypothesis has been demonstrated in a few cases where herbivores lack high quality food sources [32,35,30]. These hypotheses were tested by measuring the nutritional quality of the freshly detached algae mentioned above, and by measuring *D*. *nigerrima*’s consumption, preference, absorption and growth rates while presented with a diet of these algae.

## Material and methods

### Collection of snails and algae

**Fig 1:**
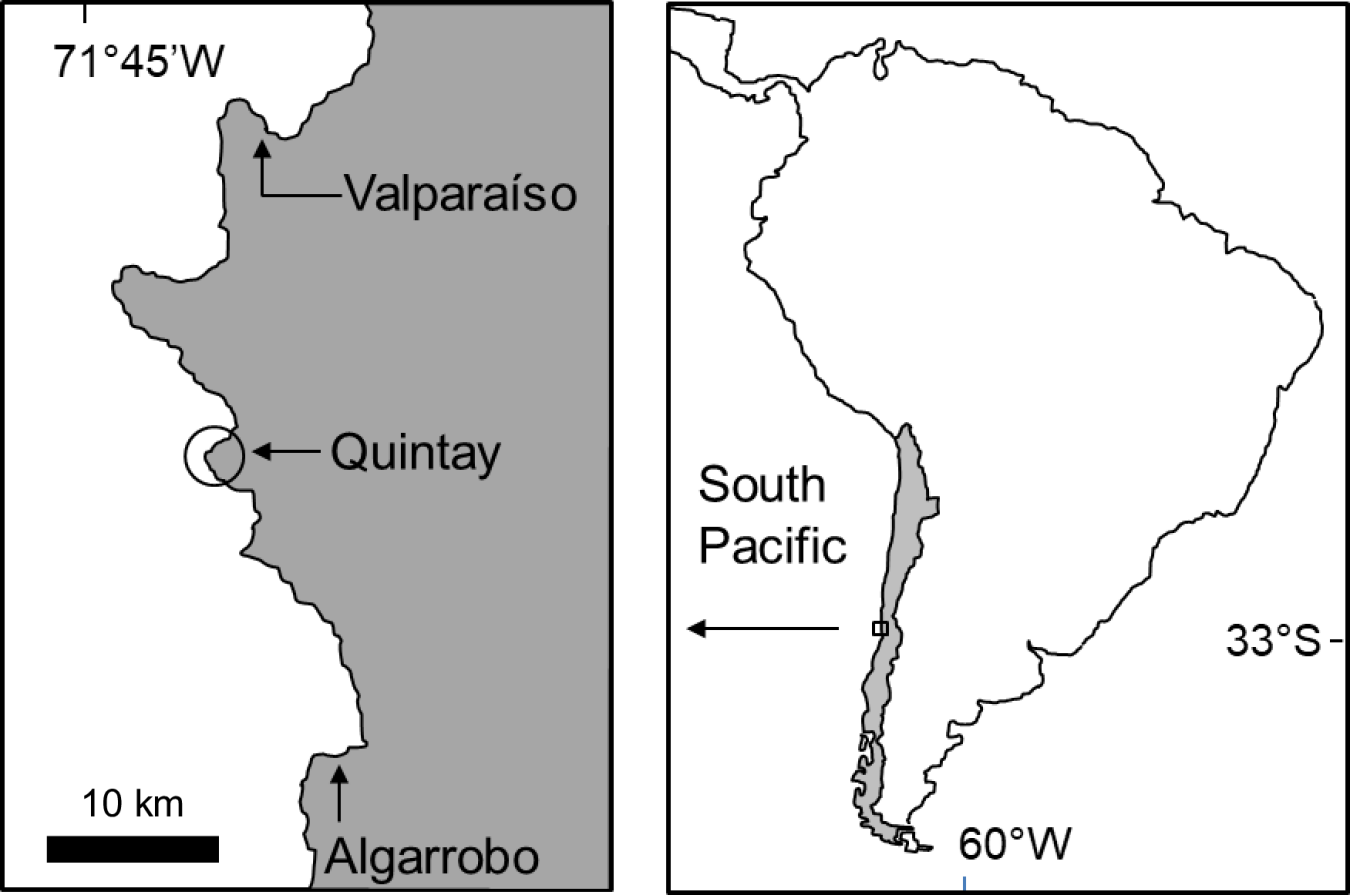
Map illustrating the approximate location of Quintay along the Chilean littoral, Southeast Pacific.

Individual snails (*D*. *nigerrima*) were manually collected from the rocky intertidal of Quintay, Central Chile (~33°11’S, 71°41’W) (Fig 1) during July 2014. The specimens were transported to the facilities of the Centro de Investigaciones Marinas de Quintay (CIMARQ; Universidad Andres Bello) and maintained in containers with filtered seawater and scattered rocks for acclimation. The containers were tightly covered with perforated lids to allow for air exchange. Prior to the experiments, the specimens were starved a standard 48 h period to standardize hunger levels and avoid the potential influence of previous *in situ* diets on subsequent feeding behavior [28,10,30]. The three species of algae (*D*. *antarctica*, *L*. *spicata* and *L*. *trabeculata*) were regularly collected from the same rocky shore area in Quintay and fresh pieces of each species were used immediately after collection for the experiments described below. Given that the focus of this study is on fresh subsidies of detached algae [10,30] no decomposing pieces were used in any of the experiments. The duration of the experiments varied according to the variables being measured (see below).

### Algal nutritional quality

The nutritional quality of fresh pieces of each species of alga was assessed in terms of organic matter and protein content. Algal samples (blades) from each species (n = 5) were dried (60°C, 48 h) and weighed and subsequently incinerated (500°C, 4 h) before being weighed again. The organic fraction of these algae was then estimated by weight loss expressed as percentage. Protein content of the algae was assessed using the bicinchoninic acid method (Pierce^TM^ BCA Protein Assay Kit) using bovine albumin serum as a standard. Samples were mixed with SDS (0.5%), sonicated for 1.5 min and centrifuged at 5,500 rpm for 35 min. The supernatant was incubated with BCA at 45°C for 30 min. Protein concentration was determined colorimetrically by measuring the absorbance at 562 nm. Estimates were based on replicate samples of *D*. *antarctica* (n = 11), *L*. *spicata* (n = 5) and *L*. *trabeculata* (n = 4). The level of replication was based on preliminary trials using each type of algae.

### Snail consumption rates and preference

Consumption rates on each species of algae were measured separately (no choice trials) in 12.0 × 9.4 × 4.3 cm height plastic containers covered with perforated lids to allow for air exchange. Each treatment had five replicates and each replicate had 25 snails and a similar (standard) amount of algae (2–7 g of *D*. *antarctica*, *L*. *spicata*, or *L*. *trabeculata)* offered *ad libbitum*. Before starting the experiments, algal pieces were gently blotted and weighed. Consumption trials lasted 24 h under controlled temperature (17°C; representative of the average water temperature in the collection area) and a natural light/dark cycle. These trials were matched with replicated controls (n = 5) with algae but not snails to calibrate for potential weight changes due to reasons other than grazing, following published methodology [36–37]. Pieces of algae in each container were weighed before and after the exposure to snails with a 0.001 mg accuracy digital balance, and algal consumption rates estimated as follows: Consumption = (Einitial—E_final_)–(C_initial_-C_final_), where E and C stand for algae exposed to snails and those used as controls, respectively.

For the assessment of snail consumption rates with choice (i.e. preference), gently blotted pieces of each type of algae were weighed (approximately 2–8 g of each algal species), and offered simultaneously to snails (1.1–1.5 mm shell length). In each replicate (n = 10), the experimental animals (40) were placed in containers (19.3 × 12.7 × 6.7 cm height) containing all three macroalgal species. All experiments were conducted for 24 h in the same controlled conditions described above. The higher number of replicates for these experiments (algal choice) followed previous observations indicating that values of consumption with choice were in general more variable than those of consumption without choice. The higher number of snails (40) per replicate accounted for differences in container size. Regardless, consumption with and without choice were in both cases calculated as consumption rates per individual snail. Each replicate was matched with parallel control containers with algae but without snails. Consumption rates upon each type of algae were then calculated by algal weight difference, following the methodology described above.

### Snail absorption efficiency and growth

Absorption efficiency was estimated from the relationship between the organic and inorganic fractions measured in the ingested algae and the fecal material [38]. This methodology assumes that only the organic fraction of the food is affected by the absorption process. Absorption efficiency (AE) was then calculated as follows: AE = ([F-E] / [1-E] × F) × 100, where F and E represent the proportion of organic matter present in food and feces, respectively. Before beginning the experiments, the animals were maintained without food for 48 h (see Duarte et al. [30]). To obtain fecal pellets, 25 snails were maintained in 11.7 × 7.2 cm plastic containers with pieces of either *D*. *antartica*, *L*. *spicata* or *L*. *trabeculata*. Each treatment (i.e. each algal species) had five replicates. After 24 h, fecal pellets were collected and frozen, while algal pieces were replaced with fresh ones, and the procedure was then repeated for the following 4 d. To quantify organic matter, food (algae) and fecal pellets were processed following the methodology described above (see algal nutritional quality).

Growth rates were measured over individual snails (1.1–1.4 cm shell length, approximately 1 g weight) fed with each species of alga separately. Snails and 4–5 g of fresh algal pieces were placed in 11.7 × 7.2 cm plastic containers (n = 10) for 11 d, replacing algal pieces with fresh ones on a daily basis. Growth rates were estimated by before-after snail weight difference.

### Data analysis

Nutritional quality, consumption, absorption and growth rate were compared using one-way ANOVAs [39]. For those analyses that detected significant differences between algae, a Tukey’s HSD *a posteriori* test was applied to identify significant differences between individual species. ANOVA assumptions of normality and homoscedasticity were assessed using Kolmogorov-Smirnov and Bartlett tests, respectively. In the case of food preference experiments, and because of their nature (algal choice), consumption rates of a given alga were not independent from the consumption rates of the other species. Differences among algae were thus assessed with a nonparametric Friedman’s test followed by pairwise comparisons [39]. All analyses were conducted using R routines.

### Ethics statement

No specific permits were required for the described laboratory experiments. The intertidal area is part of the Laboratorio de Investigaciones Marinas de Quintay of the Universidad Andres Bello and are not privately owned or designated as protected areas (reserves or parks). No protected or endangered species were involved in this study.

## Results

### Algal nutritional quality

**Fig 2:**
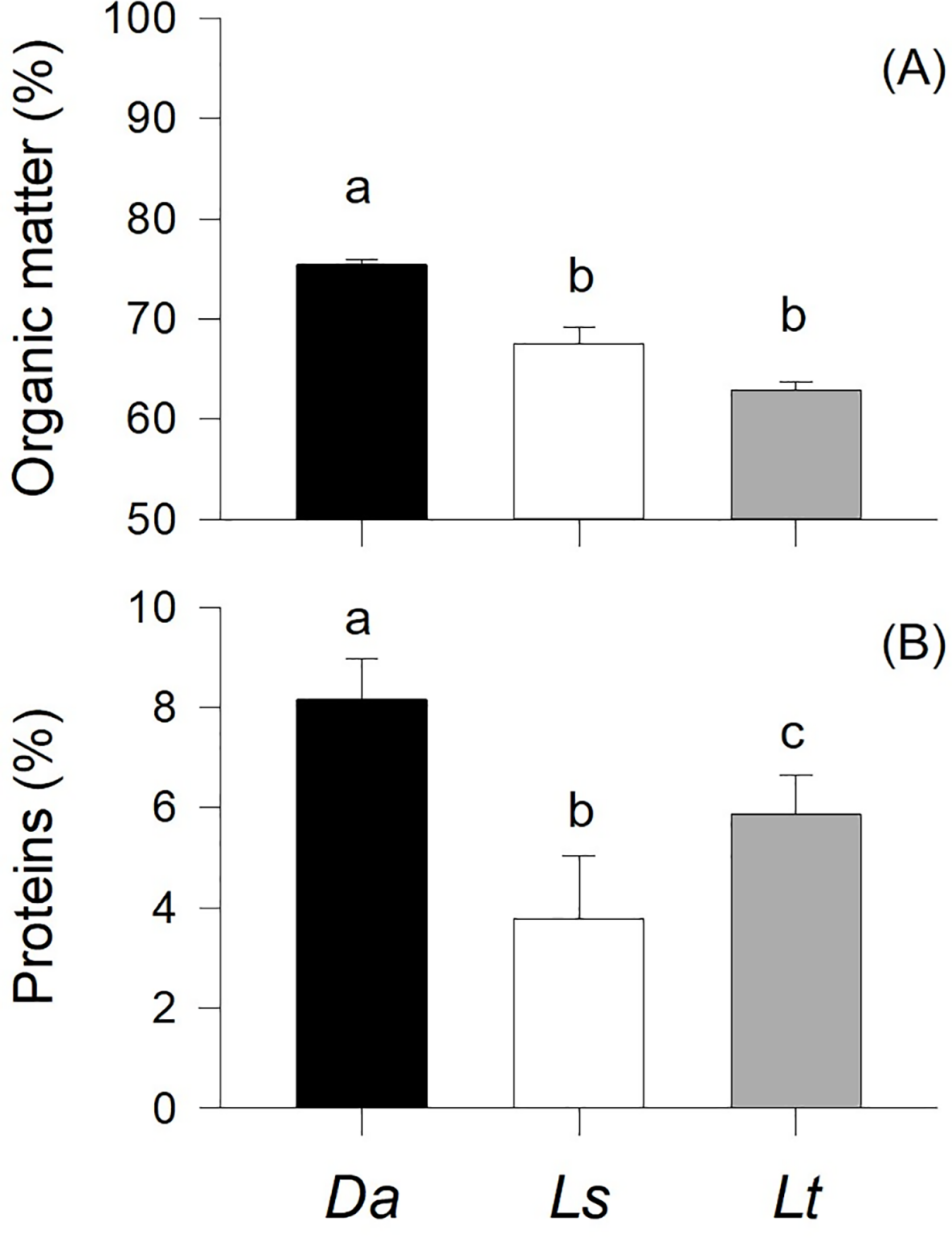
Mean (+S.D.) values of organic matter (A) and protein content (B) in the tissues of the three stranded algae consumed by *Diloma nigerrima*: *Durvillaea antarctica* (Da), *Lessonia spicata* (Ls), and *Lessonia trabeculata* (Lt). Different letters above the bars identify significant differences among means based on Tukey’s post-hoc comparisons (p<0.05).

Algal organic content differed significantly among species (p<0.001; Fig 2A) such that it was significantly higher in *D*. *antarctica* than in *L*. *trabeculata* and *L*. *spicata* (the latter two were not significantly different). Protein content also differed significantly among algae species (p<0.001; Fig 2B). *D*. *antarctica* exhibited higher protein concentrations than *L*. *trabeculata* and *L*. *spicata*. In this case, all the pairwise differences were significant (p<0.001; Fig 2B).

### Snail consumption rates and preference

**Fig 3:**
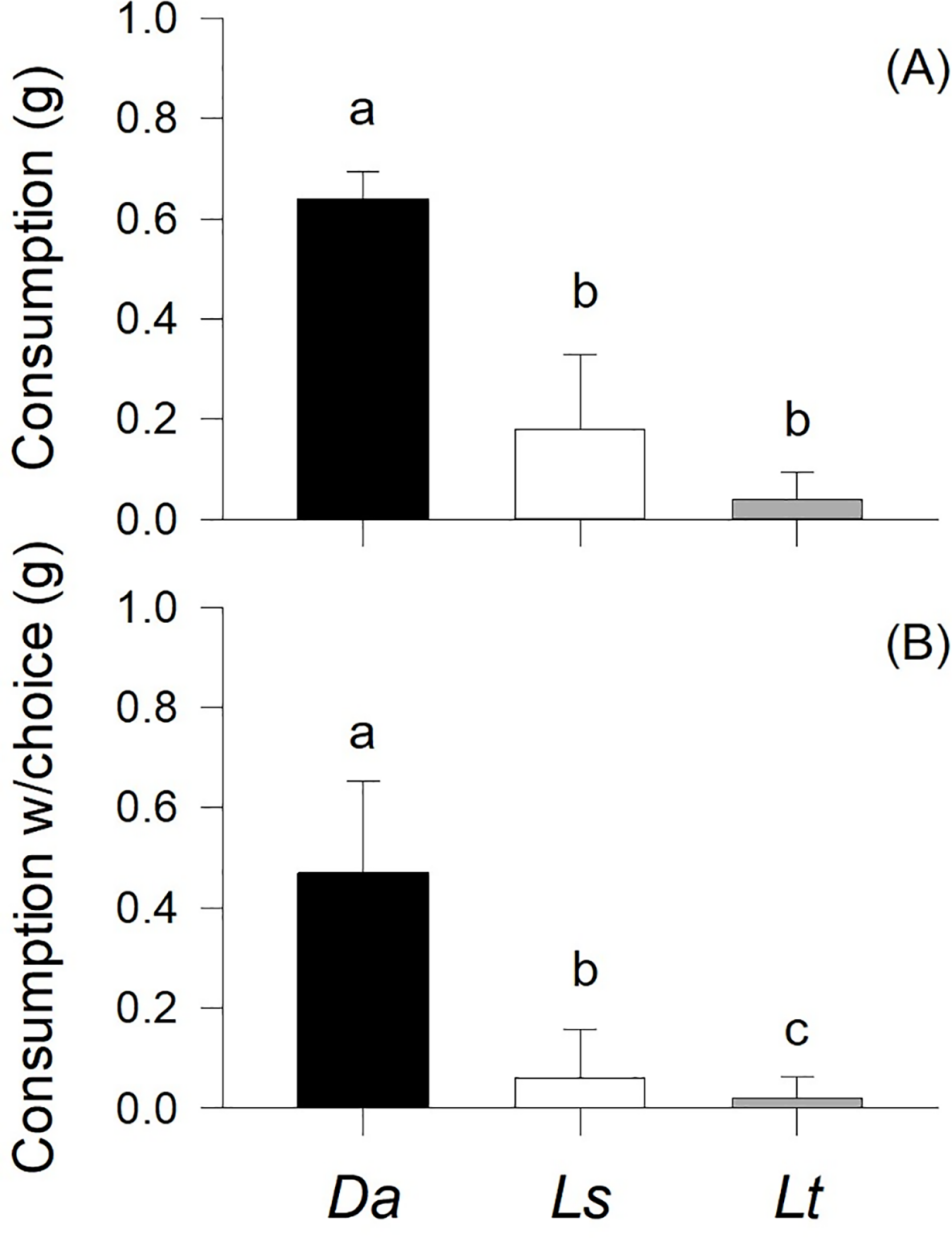
Mean (+S.D.) values of *Diloma nigerrima* consumption rates in trials without (A) and with a choice among the three species of algae (B). Different letters above the bars identify significant differences means based on Tukey’s HSD post-hoc comparisons (consumption) or Friedman’s test (consumption with choice). All other details as in Fig 2.

When algae were offered separately (i.e., no choice trials), *D*. *nigerrima* consumed significantly different amounts of algae (p<0.001; Fig 3A). Snails consumed significantly (three times) more *D*. *antarctica* than *L*. *spicata* and *L*. *trabeculata* (consumption of the latter two algae was not significantly different; p>0.05; Fig 3A). Similar results were obtained when the three algae were offered simultaneously to the snail (i.e. choice trials). In this case, *D*. *antarctica* was again the most consumed alga (p<0.001; Fig 3B), but in contrast with the experiment of consumption with no choice, *D*. *nigerrima* consumed significantly more *L*. *spicata* than *L*. *trabeculata* (p<0.05).

### Snail absorption efficiency and growth

**Fig 4:**
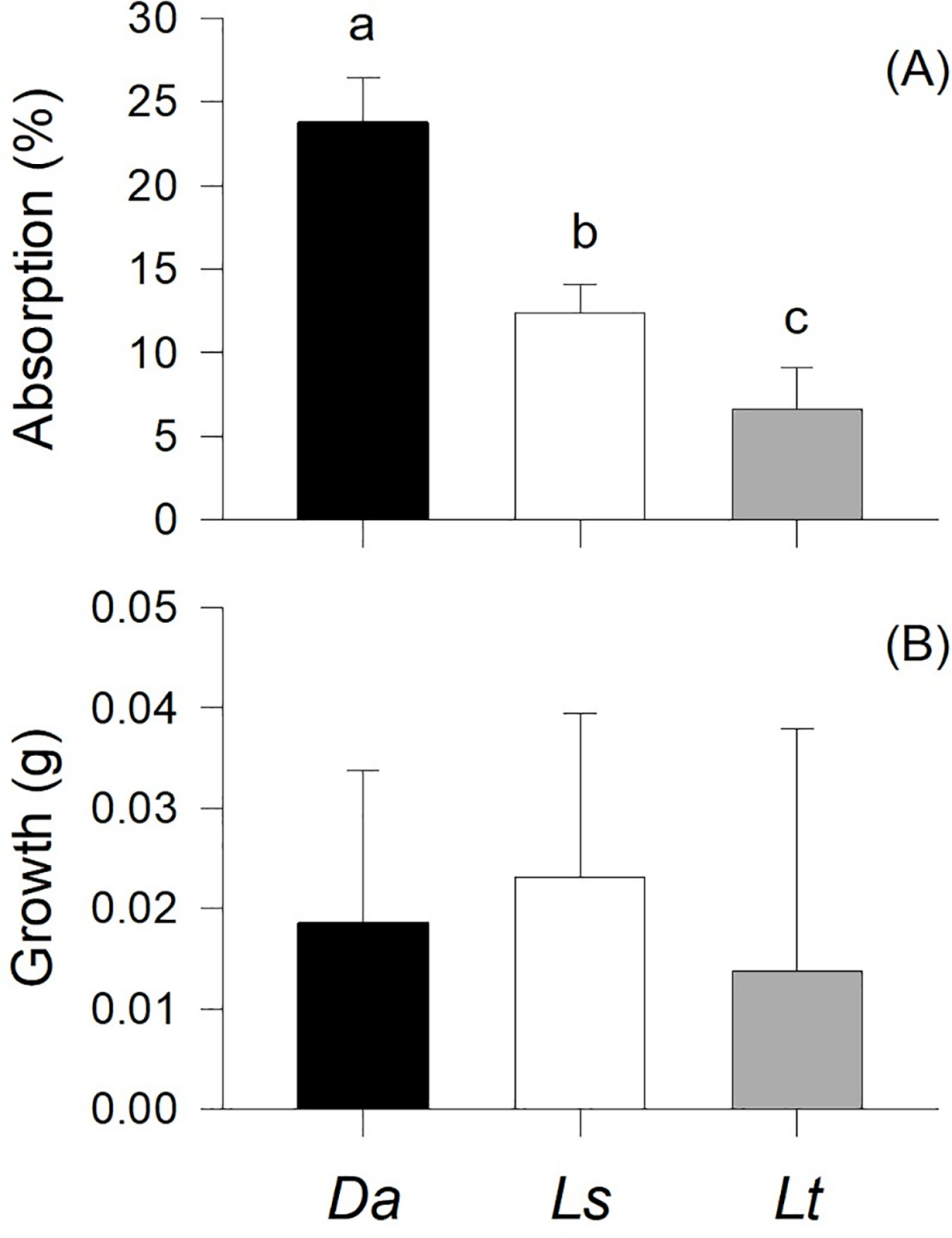
Mean (+S.D.) values of *Diloma nigerrima* absorption efficiency (A) and growth rates (B) in specimens fed separately with the three species of algae. Different letters above the bars identify significant differences means based on Tukey’s HSD post-hoc comparisons (p<0.05). The lack of letters in (B) indicates the lack of significant differences. All other details as in Fig 2.

*D*. *nigerrima*’s absorption efficiency varied significantly among algae (p<0.001) such that it was highest on *D*. *antarctica* and lowest on *L*. *trabeculata*, with intermediate values in the case of *L*. *spicata*. Snail growth rates were not significantly different among those fed with different algal species (p = 0.555; Fig 4B).

## Discussion

Herbivores relying on fresh trophic subsidies are interesting models for the study of feeding behavior for at least two reasons. First, food sources originate from a different ecosystem or ecotone [8], so their supply may be dictated by factors operating outside the habitat in which herbivory takes place. Second, in the absence of other relevant sources of food, trophic subsidies are critical and potentially drive the herbivore’s feeding behaviour, physiology, if not its survival [40,41]. As discussed below, these elements are relevant to the system studied here, where an upper intertidal snail relies on the stranding of fresh subtidal seaweeds. The bulk of the results reported here supported the predictions of the first stated hypothesis: there was a clear link between algal quality and the herbivore’s consumption and absorption efficiency, although surprisingly, that was not reflected on growth rates. The results of this study also rejected the second working hypothesis, as this herbivore was indeed able to discriminate among algal species and did not exhibit signs of compensatory feeding (*sensu* [31,35]).

### Algal quality and feeding behavior

Stranded seaweeds represent a critical source of food for various intertidal herbivore species [19,9,20,42,10]. As a result of this, the number of studies examining herbivores’ feeding on these seaweeds is growing [43,9,10,44,45]. In the system examined in the present study, the snail *D*. *nigerrima* consumed at least three times more *Durvillea antarctica* than either *Lessonia spicata* or *Lessonia trabeculata*, both in single and multiple choice algal trials. As predicted from the first hypothesis, this was directly related to algal nutritional quality [46,47,29,48,49,35]. Measurements of organic matter and protein content indicated that *D*. *antarctica* was a better quality food item than the two species of *Lessonia*, which translated on much higher feeding rates on the former species. This is consistent with the findings of Donald et al. [50], who reported the temporal disappearance of *D*. *nigerrima* populations from South African habitats that had become devoid of *D*. *antarctica*.

Among nutrient indicators, the protein content of has been considered as one of the best surrogates of algal quality for herbivores [51,52,29,30], including gastropods [53,29], amphipods [54,22,10] and echinoderms [55]. In this study, protein concentration in *D*. *antarctica* was ~25% and ~50% higher than that in *L*. *trabeculata* and *L*. *spicata*, respectively. Not surprisingly, proteins and organic matter contents were also related to *D*. *nigerrima*’s absorption efficiency rates. In fact, the variation in organic matter content across different species of seaweeds was proportionally (and almost perfectly) mirrored by the absorption efficiency measured in the snails. Such tight plant-herbivore relationships have been described before for other species [56,57,35], and associated to various measures of fitness, including herbivore growth rates [29,10,58].

Higher growth rate resulting from consumption of better quality algae has been documented in polychaetes [51], echinoderms [59–61], amphipods [10,30,58] and gastropods [29]. However, this did not occur in this study: despite the snail’s higher consumption, preference, and absorption efficiency on the most nutritious alga (*D*. *antarctica*), snails that fed upon a diet of that species alone did not grow faster or larger than those fed on either species of *Lessonia*. One plausible hypothesis to explain the lack of differences in growth rates is a potential increase in oxygen uptake while consuming *D*. *antartica*. If *D*. *nigerrima* consumes more metabolic energy while processing the most nutritious alga, this may balance its growth with the one achieved with the less nutritional algae, as could be the case in this study. Interactions with other structural features (shape and toughness) [46,62–64] or the presence of chemical defenses against herbivores [29,65,66] are also possible. The assessment of all those factors was beyond the scope of this study, but their analysis may guide further research on the species studied here.

The temperature used in the experiments (17°C) was lower than the one used in other studies (e.g. 20°C in amphipod growth trials) [10,30,58]. Although those 3°C can admittedly make a difference in growth rates, the temperature used here was based on what has been measured in the field and thus it better reflects the natural conditions of the habitat in which snails and algae are found. The replication and the duration of the trials (11 d) was judged suitable to accurately measure growth rates in *D*. *nigerrima* based on repeated observations conducted prior to the trials reported here. Hence, based on their knowledge of the species and the system, the authors are confident that the lack of differences reported here is meaningful and informative. However, depending on logistic feasibility, further studies should increase replication and/or duration of the trials to verify whether the snails reach a point at which growth rates become different.

### Lack of compensatory feeding and further directions

In marine invertebrates, particularly amphipods, food preference has been directly correlated with growth rates or other measures of fitness [67,68,10,29,45]. This was not the case in this study, where the clear preference of *D*. *nigerrima* for a particular alga (*D*. *antarctica*) was not matched by enhanced growth rates when the snail was fed on a diet of that species. When no direct relationship emerges between food preference and a measure of fitness, such as growth, as in this study, an examination of individual consumption rates may shed light on other feeding strategies. One of these strategies is compensatory feeding [31,35] or the increased consumption of food items of comparatively lower nutritional quality in order to achieve optimal growth in the absence of better quality food. However, in no-choice consumption trials, *D*. *nigerrima* exhibited the same patterns of consumption as in preference trials, indicating the absence of evidence of compensatory feeding. Other species are able to compensate by balancing growth with increased absorption efficiency of lower quality algae [30,58,31,35]. However, this was not the case either as the snail’s absorption rates were again significantly higher on the preferred species, *D*. *antarctica*.

The second hypothesis was therefore firmly rejected given the identification of a clear preference for one algal species and the absence of evidence of compensatory feeding. In the search for alternative explanations, the physical and nutritional conditions of the seaweeds must be considered. Freshly detached algae (the focus of this study) that have been “uprooted” from their original subtidal habitat may become deposited in a harsher (less productive) upper-intertidal environment by the virtue of the connectivity between ecosystems [69,70]. The fact that a fraction of those algae remains stranded over the intertidal rocks and undergo decay for an uncertain number of days cannot be ignored. The condition of these algae may vary widely and have an effect on herbivores’ preferences and consumption [41,57]. Hence, even though the analysis of decomposing algae is well beyond the scope of this study, it is wise to suggest further studies addressing the role of algal decay on the feeding ecology of *Diloma nigerrima*.

Together, the results of this study clearly show that *D*. *nigerrima* is able to discriminate among the three stranded seaweeds, as has been demonstrated for other marine herbivores. Furthermore, the hypothesized increase in oxygen uptake while consuming *D*. *antarctica* is a plausible explanation for the mismatch between food preference and growth recorded here. The differences in seaweed quality and snail’s feeding responses are accurate and meaningful but, as previously indicated, do not exclude alternative factors interacting with the variables measured here [29,46,62–66]. For upper intertidal herbivores relying on the input of freshly detached seaweeds from other ecotones, further study of multiple factors affecting their feeding strategies may become central to the understanding of herbivore-seaweed relationships.
